# Endoplasmic reticulum stress inhibits 3D Matrigel‐induced vasculogenic mimicry of breast cancer cells via TGF‐β1/Smad2/3 and β‐catenin signaling

**DOI:** 10.1002/2211-5463.13259

**Published:** 2021-08-19

**Authors:** Huifen Liu, Hao Wang, Dan Chen, Cuirong Gu, Jianming Huang, Kun Mi

**Affiliations:** ^1^ Radiation Oncology Key Laboratory of Sichuan Province Sichuan Cancer Hospital & Institute Sichuan Cancer Center School of Medicine University of Electronic Science and Technology of China Chengdu China; ^2^ Breast Surgery Sichuan Cancer Hospital & Institute Sichuan Cancer Center School of Medicine University of Electronic Science and Technology of China Chengdu China

**Keywords:** breast cancer, endoplasmic reticulum stress, TGF‐β1, vasculogenic mimicry, β‐catenin

## Abstract

Endoplasmic reticulum (ER) stress is a cellular stress condition involving disturbance in the folding capacity of the ER caused by endogenous and exogenous factors. ER stress signaling pathways affect tumor malignant growth, angiogenesis and progression, and promote the antitumor effects of certain drugs. However, the impact of ER stress on the vasculogenic mimicry (VM) phenotype of cancer cells has not been well addressed. VM is a phenotype that mimics vasculogenesis by forming patterned tubular networks, which are related to stemness and aggressive behaviors of cancer cells. In this study, we used tunicamycin (TM), the unfolded protein response (UPR)‐activating agent, to induce ER stress in aggressive triple‐negative MDA‐MB‐231 breast cancer cells, which exhibit a VM phenotype in 3D Matrigel cultures. TM‐induced ER stress was able to inhibit the VM phenotype. In addition to the tumor spheroid phenotype observed upon inhibiting the VM phenotype, we observed alterations in glycosylation of integrin β1, loss of VE‐cadherin and a decrease in stem cell marker Bmi‐1. Further study revealed decreased activated transforming growth factor β1, Smad2/3, Phospho‐Smad2 and β‐catenin. β‐Catenin knockdown markedly inhibited the VM phenotype and resulted in the loss of VE‐cadherin. The data suggest that the activation of ER stress inhibited VM phenotype formation of breast cancer cells via both the transforming growth factor β1/Smad2/3 and β‐catenin signaling pathways. The discovery of prospective regulatory mechanisms involved in ER stress and VM in breast cancer could lead to more precisely targeted therapies that inhibit vessel formation and affect tumor progression.

## Graphical abstract

Tunicamycin‐induced endoplasmic reticulum stress inhibited the VM phenotype of breast cancer cells in 3D Matrigel cultures. Cells exhibited tumor spheroid phenotype, alterations in glycosylation of integrin β1, loss of VE‐cadherin and a decrease in stemness. Further study revealed decreased levels of activated transforming growth factor β1, Smad2/3, Phospho‐Smad2 and β‐catenin, suggesting that activation of endoplasmic reticulum stress inhibited the VM phenotype via both the transforming growth factor β1/Smad2/3 and β‐catenin signaling pathways.

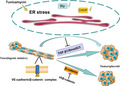

**Abbreviations**
3DM: 3D Matrigel culture
CCK‐8: Cell Counting Kit‐8
ECM: extracellular matrix
EMT: epithelial–mesenchymal transition
ER: endoplasmic reticulum
IC_50_
: half‐maximal inhibitory concentration
PAS: periodic acid–Schiff
shRNA: short hairpin RNA
TGF‐β1: transforming growth factor β1
TM: tunicamycin
TNBC: triple‐negative breast cancer
VM: vasculogenic mimicry

Vasculogenic mimicry (VM) refers to a phenotype of aggressive tumor cells that mimic embryonic vasculogenesis by forming patterned tubular networks in the tumor extracellular matrix (ECM) lined with glycogen‐rich molecules and basement membrane proteins [1]. It is a potential transdifferentiation event that suggests a unique capability of certain aggressive tumor cells related to epithelial–mesenchymal transition (EMT) and stemness [2, 3, 4, 5]. VM has been reported to be associated with poor prognosis, tumor metastasis and drug resistance in several tumors, including breast cancer [6]. The underlying molecular pathways supporting VM are associated with vascular, embryonic and/or stem cell, and hypoxia‐related signaling pathways [7]. The formation of VM phenotype involves the interaction of integrins with ECM components [8], cytoskeleton reorganization and activation of signaling, including FAK signaling [9], c‐myc/snail/Bax signaling [10], PI3K/Akt/mTOR signaling [11], transforming growth factor β1 (TGF‐β1)/Smad2/3 signaling [12], Wnt/β‐catenin signaling [13] and β‐catenin/Tcf4 signaling [14]. Whether there are other factors involved in the regulatory mechanism of VM and crosstalk among these signaling molecules remains elusive.

Endoplasmic reticulum (ER) is a central cellular organelle with important functions, such as synthesis, folding and posttranslational modifications of proteins [15]. A variety of endogenous and exogenous factors could cause the accumulation of unfolded or misfolded proteins in the ER lumen, which results in ER stress and activates the UPR process [16, 17]. Accumulating evidence indicates that ER stress pathways evocation could promote the antitumor effects of certain agents and drug candidates [18, 19, 20]. ER stress is also involved in the processes of cellular interactions with the tumor microenvironment, such as immune modulation and inflammation [21, 22], and the key ER stress sensor IRE1α signaling pathway has been reported to affect tumor malignant growth, angiogenesis and progression via the microenvironment directly and indirectly [23, 24, 25]. Hypoxia could induce miR‐153 expression via triggering ER stress, and miR‐153 directly inhibited expression of the hypoxia‐inducible factor 1‐alpha and suppresses breast cancer angiogenesis by decreasing the secretion of vascular endothelial growth factor A (VEGFA) [24]. However, the relationships between ER stress and VM remain undetermined, and which markers and signaling pathways involved in the effect of ER stress on VM have not been investigated in cancers that need to be further explored. Thus, we decided to explore their relationship and mechanism for the first time in triple‐negative breast cancer (TNBC), which is a highly aggressive malignancy with poor posttreatment prognosis.

In this study, we used 3D Matrigel culture (3DMs) model to establish the VM phenotype of TNBC cells. After using tunicamycin (TM), an ER stress‐inducing agent that activates UPR, we found that TM inhibited the VM phenotype of TNBC cells via both the TGF‐β1/Smad2/3 and β‐catenin signaling pathways. The results might provide a new perspective of novel antiangiogenic strategy that could complement the insufficient efficacy of antiangiogenic therapy in TNBC.

## Materials and methods

### Reagents and antibodies

TM was purchased from Cell Signaling (Danvers, MA, USA). Matrigel and collagen type I were purchased from Corning (Bedford, MA, USA). Human TGF‐beta1 Quantikine ELISA Kit was purchased from R&D Systems (Minneapolis, MN, USA). DAPI and pyrvinium were purchased from Sigma (St. Louis, MO, USA). Phalloidin was purchased from Yuheng Biotechnology (Suzhou, China). Glycogen Periodic Acid Schiff stain kit was purchased from Solarbio Life Sciences. Puromycin was purchased from BioFroxx (Guangzhou, China). X‐tremeGENE transfection reagent was purchased from Roche (Mannheim, Germany). Antibodies used were as follows: human CD44–FITC and CD24–phycoerythrin and their respective isotype controls were obtained from BD Biosciences (Franklin Lakes, NJ, USA). β‐Catenin was purchased from Santa Cruz (Dallas, TX, USA). VE‐cadherin, integrin β1, BiP, CHOP, Smad2/3, Phospho‐Smad2, Phospho‐β‐catenin (Ser33/37/Thr41) and Phospho‐β‐catenin (Ser675) were purchased from Cell Signaling. Bmi‐1 was purchased from Epitomics (Burlingame, CA, USA). Short hairpin RNA (shRNA) encoding β‐catenin and scramble control shRNA were purchased from TsingKe Biological Technology (Chengdu, China).

### Cell culture

Breast cell lines were obtained from the National Collection of Authenticated Cell Cultures (Shanghai, China). For 3D cultures, cells cultured on plastic were trypsinized, washed and pelleted by centrifugation. The 3DM gels were prepared according to the manufacturer's instructions with a final concentration of 5 mg·mL^−1^. Experiments were carried out in 24‐well plates using Matrigel and single‐cell suspensions. Whenever coculture experiments were performed, the interacting cells were premixed in equal numbers in suspension before embedding in gels. After 30‐min incubation at 37 °C, the gelled block of cells was overlaid with the culture medium, subsequently changed every 2 days. For 3D collagen cultures, the gels were prepared according to the manufacturer's instructions with a final concentration of 1.5 mg·mL^−1^.

### Immunostaining

The morphology immunofluorescence was examined by working solution of phalloidin (5 U·mL^−1^) and DAPI (300 nm), which were diluted in PBS buffer for F‐actin and nuclear staining, respectively. Immunofluorescent images were obtained with laser confocal microscopy Nikon A1 (Nikon Corporation, Tokyo, Japan). A confocal z‐stacking program was used to achieve series of optical sections and rebuild 3D images by NIS‐Elements AR Analysis (Nikon Corporation).

### Flow cytometry

Combinations of fluorochrome‐conjugated monoclonal antibodies against human CD44–FITC and CD24–phycoerythrin or their respective isotype controls were added to the cell suspension at concentrations recommended by the manufacturer and incubated at 4 °C in the dark for 30 min. The labeled cells were analyzed on a BD Canto Ⅱ flow cytometer.

### Periodic acid–Schiff staining

3DM cells were fixed at 4% paraformaldehyde in PBS for 20 min at room temperature; then tubular networks in Matrigel were stained with periodic acid–Schiff (PAS) staining kit according to the manufacturer's instructions followed by several washes with PBS at room temperature.

### TM treatment

3D cultured cells were treated with different concentrations of TM for 48 h and then tested for viability by Cell Counting Kit‐8 (CCK‐8) assays. CCK‐8 was purchased from Beijing 4A Biotech Company (Beijing, China). After treatment of TM, MDA‐MB‐231 cells were analyzed for phenotypes by immunostaining and protein expression by western blots. CCK‐8 assays were performed to determine the cells' half‐maximal inhibitory concentrations (IC_50_) to TM for β‐catenin knockdown and control groups, respectively.

### TGF‐β1 ELISA

The concentration of activated TGF‐β1 was determined using supernatants from 3DMs before and after TM treatment by the Human TGF‐beta1 Quantikine ELISA Kit (R&D Systems) following the manufacturer's protocol.

### Western blots

Proteins were extracted from cells using radioimmunoprecipitation assay buffer and protease–phosphatase inhibitor cocktails; then insoluble material was removed by centrifugation at 22 000 ***g*** for 30 min. Protein electrophoresis was performed by standard SDS/PAGE methods, using reducing sample buffer. Proteins were transferred to poly(vinylidene difluoride) membranes and blocked in 5% nonfat dried milk in TBST buffer. All of the antibodies were diluted in the blocking buffer. Membranes were washed in TBST buffer. Antibody binding was detected using secondary antibodies and the SuperSignal chemiluminescent substrate, according to the manufacturer's instructions.

### Lentivirus‐mediated RNA interference

For β‐catenin knockdown, lentiviruses encoding β‐catenin shRNA or scramble control shRNA were produced by 293T cells. Cells were infected with control shRNA lentivirus or β‐catenin shRNA lentiviruses for 24 h. Positive clones expressing shRNAs were selected with puromycin (2 μg·mL^−1^). Western blot analysis was used to determine the expression levels of β‐catenin in these cells.

### Migration assays

Different groups of cells (2 × 10^5^/well) were seeded in six‐well plates and grown to about 80% confluence. Then cells were scraped with 1000‐μL sterile micropipette tip to create a wound. The gap was photographed at first and after 20 h, and the gap filling was photographed.

### Statistical methods

Statistical significance was determined for experimental data by using the Student's *t*‐test. Error bars are representative of the standard error of mean of three biological experiments in all cases. A *P* value <0.05 was regarded as statistically significant (*P < 0.05).

## Results

### VM and tumor spheroid phenotypes of breast cancer cells in 3DMs

In this study, we examined the phenotype of several human breast cancer cell lines with different aggressive properties (MDA‐MB‐231, BT‐549, MDA‐MB‐435S and MCF‐7 cells) and one nonmalignant breast cell (MCF 10A) in 3DMs. The results showed that both MDA‐MB‐231 and BT‐549 cells could form tubular networks in 3DMs, while other cells formed tumor spheroids instead (Fig. S1A). MDA‐MB‐231 and BT‐549 cells have been identified as TNBC cells, one kind of highly aggressive breast cancer cell that is more prone to form VM in Matrigel culture. Because up‐regulation of the endothelial marker VE‐cadherin has been considered as the key event in VM formation [26], we then examined the VE‐cadherin expression in the tubular networks of MDA‐MB‐231 and BT‐549 cells in 3DMs by western blots. The results indicated that only tubular networks formed by MDA‐MB‐231 cells expressed VE‐cadherin, which could be considered as VM phenotype (Fig. S1B). Therefore, our results in the following parts were derived mainly from the aggressive triple‐negative MDA‐MB‐231 cells in 3DM.

**Fig. 1:**
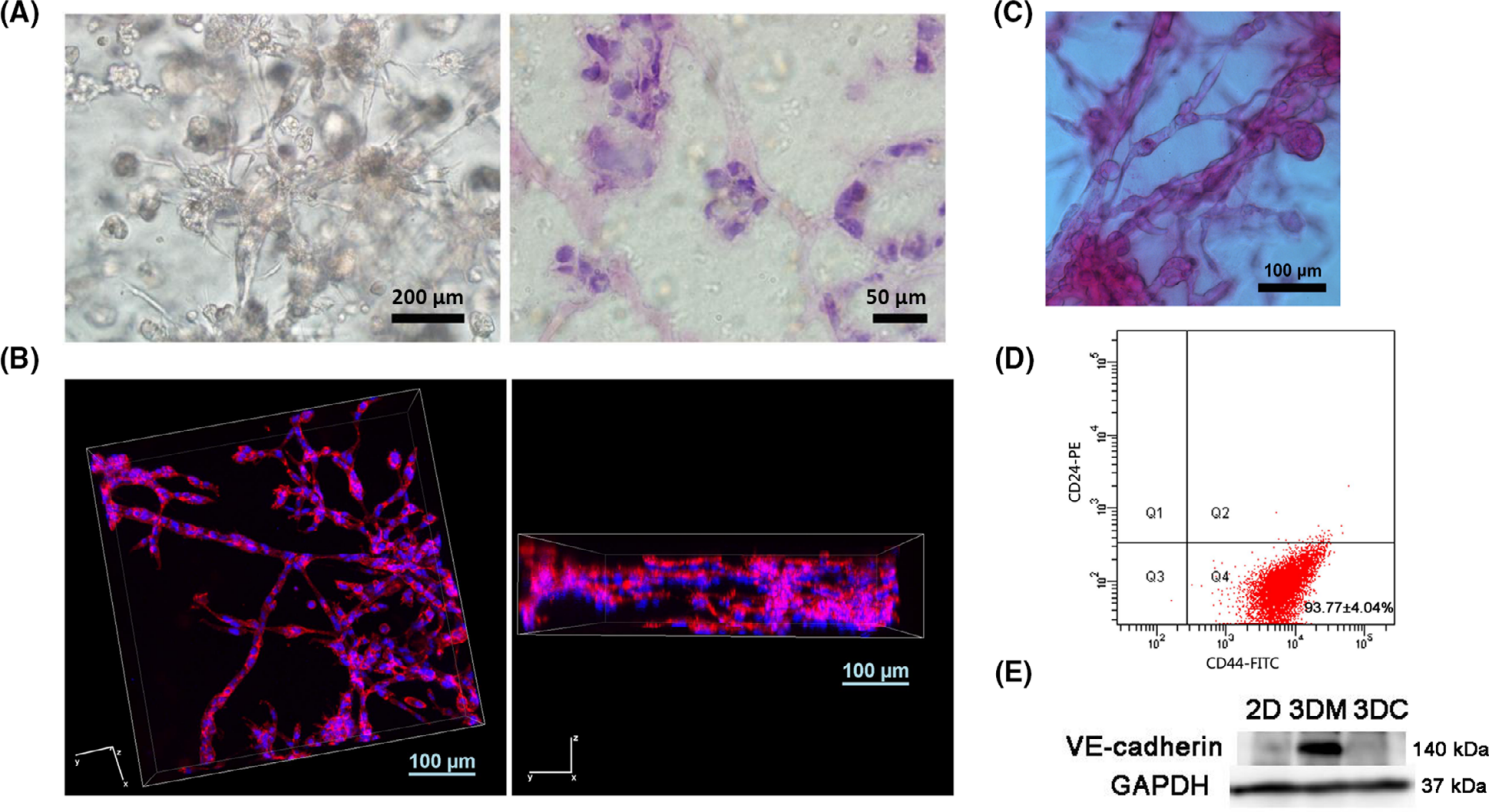
VM formation of MDA‐MB‐231 breast cancer cells in 3DMs. (A) Morphology of MDA‐MB‐231 breast cancer cells in 3DMs by light microscope (left; scale bar: 200 μm) and hematoxylin and eosin staining (right; scale bar: 50 μm). (B) 3D reconstruction images of VM phenotype of MDA‐MB‐231 cells in 3D cultures by using a confocal z‐stacking program. Scale bars: 100 μm. (C) PAS staining image of VM phenotype of MDA‐MB‐231 cells in 3D culture. Scale bar: 100 μm. (D) Flow cytometric analysis of surface markers CD24 and CD44 in MDA‐MB‐231 cells. (E) Western blot analysis of VM marker protein VE‐cadherin in MDA‐MB‐231 cells from 2D Matrigel culture, 3DM, and 3D collagen culture (3DC) groups.

3DM MDA‐MB‐231 cells formed tubular net‐like morphology, which was identified by light microscopy and the hematoxylin and eosin staining of cryostat sections of the 3D scaffold (Fig. 1A). Moreover, the localization of F‐actin and DAPI in the networks at the endpoint of 5 days was examined by confocal microscopy (Fig. 1B). We used a confocal z‐stacking program to achieve series of optical sections and rebuild 3D images by NIS‐Elements AR analysis to reveal the 3D structural construction of VM phenotype of MDA‐MB‐231 cells. We next performed PAS staining to identify glycogen and related mucopolysaccharides secreted by MDA‐MB‐231 cells in 3D Matrigel, which exhibited strong PAS positivity (Fig. 1C). Flow cytometry was used to assess the expression of putative breast cancer stem cell markers of CD44/CD24 in MDA‐MB‐231 cells and displaying a proportion of 93.77% ± 4.04% of the CD44^+^/CD24^−^ subpopulation (Fig. 1D). The results indicated that 3DMs could mimic the ECM microenvironment *in vivo* and promote the formation of VM phenotype in CD44^+^/CD24^−^‐enriched TNBC MDA‐MB‐231 cells, hence enhancing the aggressive behaviors of these cells. Compared with 2D control and another 3D collagen culture model that could not form VM phenotype in which cells exhibited scattered single spindle‐like morphology (Fig. S1C), VE‐cadherin expressed only in MDA‐MB‐231 cells with VM phenotype in 3DMs (Fig. 1E).

### Phenotype changes after TM treatment

TM, a N‐glycosylation inhibitor, is a nucleoside antibiotic that inhibits the first step in the cellular biosynthesis of N‐linked oligosaccharides. As a result, the glycoproteins could not be folded correctly and cause an accumulation of misfolded or unfolded glycoproteins in the ER, resulting in ER stress and activation of the UPR [27, 28, 29]. At first, we sought to determine the optimal concentration of TM for treatment without excessive cell death. We treated MDA‐MB‐231 cells with different doses of TM for 48 h and assessed cell viability. The results showed 200 ng·mL^−1^ TM induced 30% cell death, and we used the concentration for our next experiments.

**Fig. 2:**
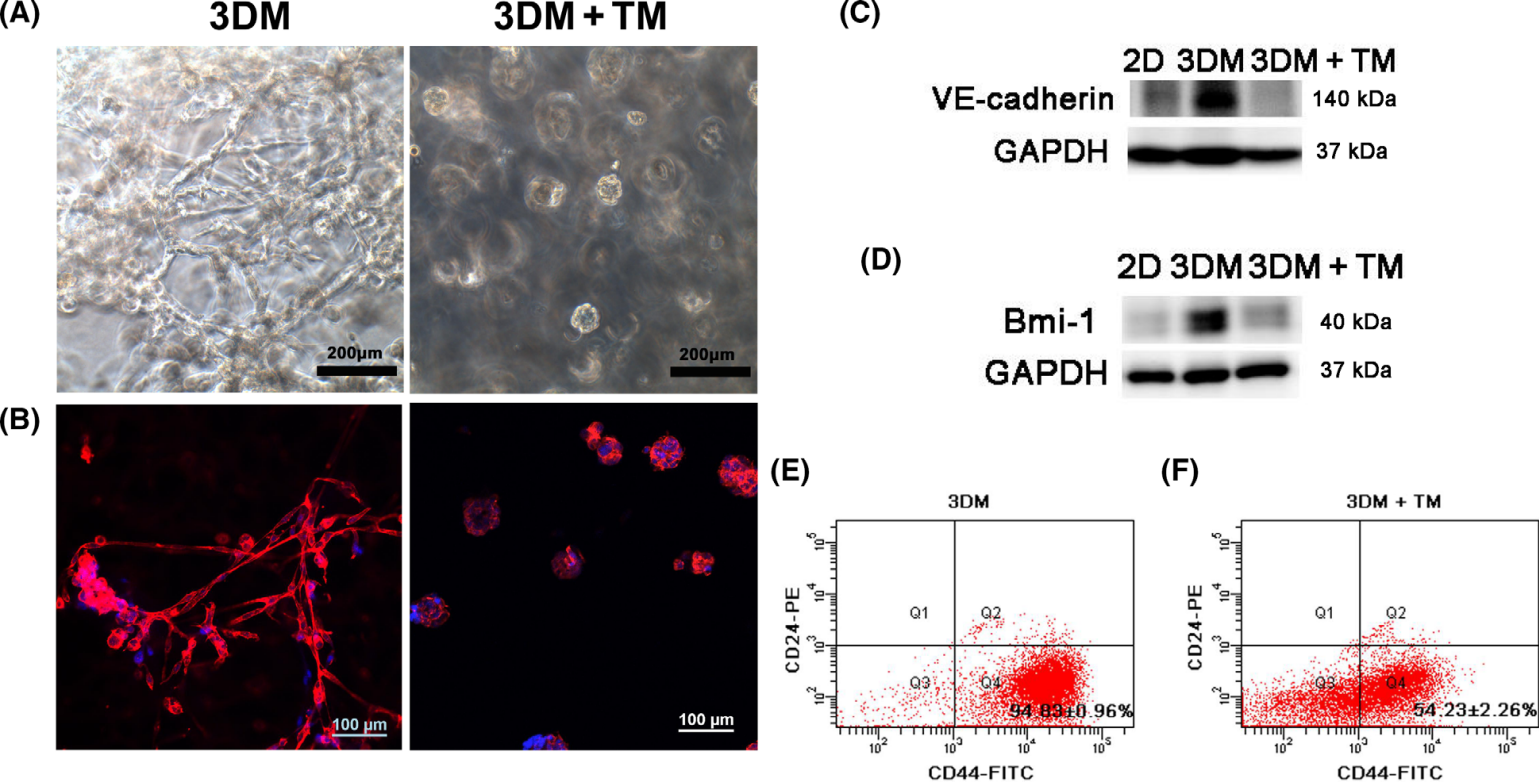
Phenotypic changes of MDA‐MB‐231 cells in 3DM after TM treatment. (A) Light microscope images of MDA‐MB‐231 cells in 3DM and TM‐treated 3DM (3DM+TM). Scale bars: 200 μm. (B) F‐actin (red) and nuclear (blue) DAPI fluorescence images of MDA‐MB‐231 cells in 3DM and 3DM+TM. Scale bars: 100 μm. (C, D) Western blot analysis of VM marker protein VE‐cadherin and stem cell marker Bmi‐1 of MDA‐MB‐231 cells in 2D, 3DM and 3DM+TM. (E) Flow cytometric analysis of surface markers CD24 and CD44 in 3DM MDA‐MB‐231 cells. (F) Flow cytometric analysis of surface markers CD24 and CD44 in 3DM MDA‐MB‐231 cells after TM treatment.

After TM treatment, 3DM MDA‐MB‐231 cells formed tumor spheroid morphology instead of tubular networks (Fig. 2A,B). The spheroid phenotype resembled the phenotype of epithelial cell lines of MCF‐7 and MCF 10A in 3DMs. Meanwhile, the expressions of VE‐cadherin and stem cell marker Bmi‐1 were significantly down‐regulated as shown by the western blot results (Fig. 2C,D). Flow cytometry data showed that compared with the 3DM group with a proportion of 94.83% ± 0.96% of CD44^+^/CD24^−^ subpopulation cells (Fig. 2E), cells treated with TM in the 3DM+TM group exhibited a proportion of 54.23% ± 2.26% (Fig. 2F), which was significantly decreased compared with the 3DM group. The results presented here suggest that TM inhibited the VM phenotype of MDA‐MB‐231 cells as evidenced by morphology changes, down‐regulation of VE‐cadherin and Bmi‐1, and decrease of CD44^+^/CD24^−^ subpopulation cells.

### Changes in glycosylation of integrin β1 after TM treatment

**Fig. 3:**
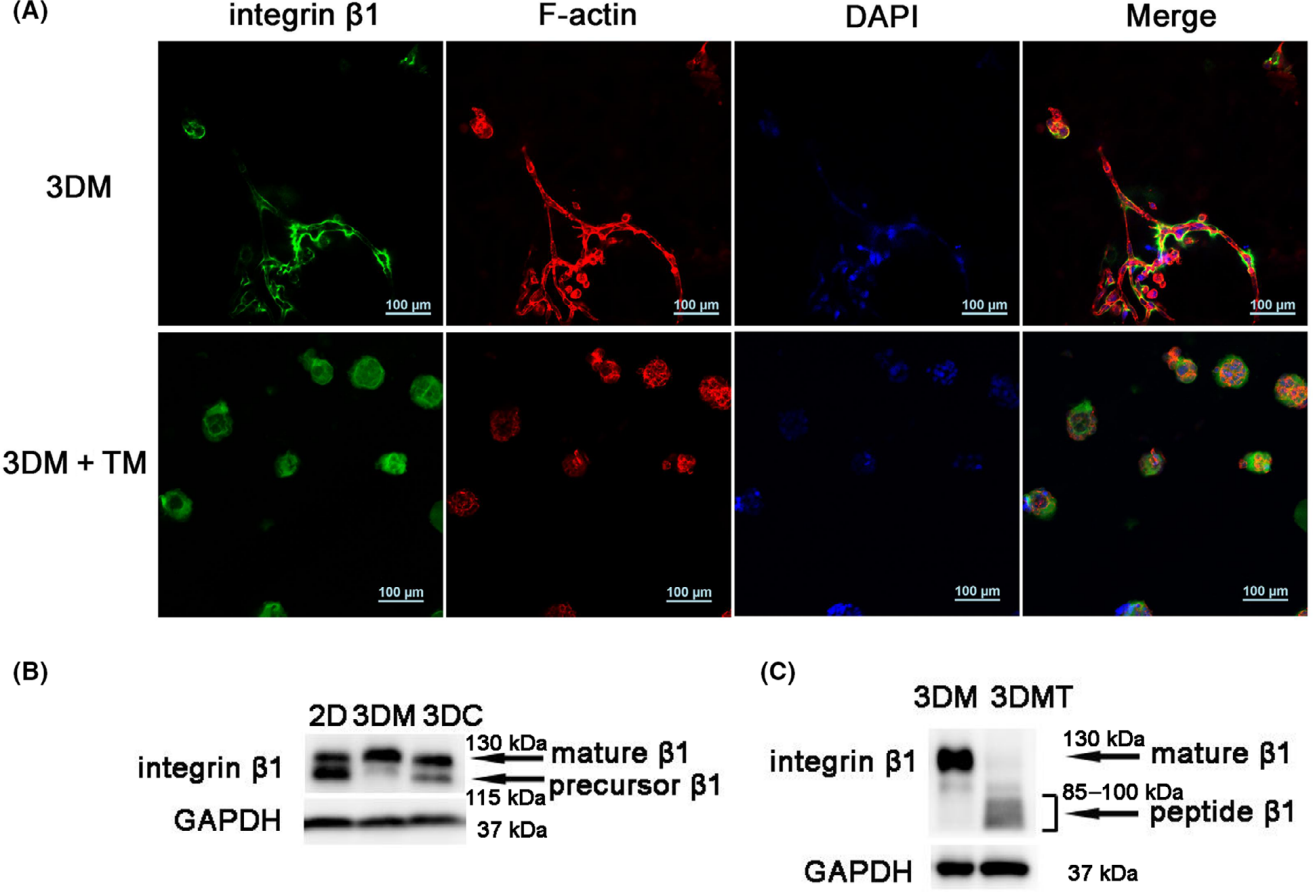
Changes in glycosylation of integrin β1 after TM treatment. (A) Integrin β1 (green), F‐actin (red) and nuclear (blue) DAPI fluorescence images and merged images of MDA‐MB‐231 cells in 3DM and TM‐treated 3DM (3DM+TM). Scale bars: 100 μm. (B) Western blot analysis of integrin β1 in MDA‐MB‐231 cells from 2D, 3DM and 3D collagen culture (3DC) groups. (C) Western blot analysis of integrin β1 in MDA‐MB‐231 cells before (3DM) after TM treatment (3DMT).

There are two stages in integrin β1 maturation, one is the glycosylation of the 86‐kD core peptide, which is a TGF‐β1‐independent process. The second is TGF‐β1‐mediated conversion of the 115‐kD integrin β1 precursor into the mature 130‐kD form [30]. The localization of integrin β1 in MDA‐MB‐231 cells before and after TM treatment was studied by immunostaining. It has been reported that mature integrin β1, but not integrin β1 precursor, could be transported with integrin α to the cell surface so that the regulation of the maturation of integrin β1 may control cell–ECM or cell–cell adhesion and other cellular processes [31, 32]. Fluorescent images of MDA‐MB‐231 cells stained with integrin β1 antibody demonstrated that in VM phenotype, integrin β1 was mainly located on the cell surface. Although after TM treatment integrin β1 was distributed throughout the cell (Fig. 3A), the glycosylation state of integrin β1 in MDA‐MB‐231 cells before and after TM treatment was studied by western blots. As shown in Fig. 3B, integrin β1 in 3DM cells with VM phenotype exhibited a significant increase of the more fully glycosylated mature form of 130 kD and loss of partially glycosylated precursor form of 115 kD, while the majority of integrin β1 in the 2D cultured cells was in its 115‐kD precursor form. We introduced another 3D culture model of collagen Ⅰ for 3D culture control. Although there was an increase in the mature form of 130 kD, the precursor form of 115 kD still existed in 3D collagen cultured cells. Cells in Matrigel had the fullest glycosylated mature form compared with other controls. After TM treatment, western blot results indicated a series of bands of incompletely glycosylated peptide of the integrin β1, and the glycosylation status of the integrin β1 was changed (Fig. 3C).

### ER stress induction after TM treatment

**Fig. 4:**
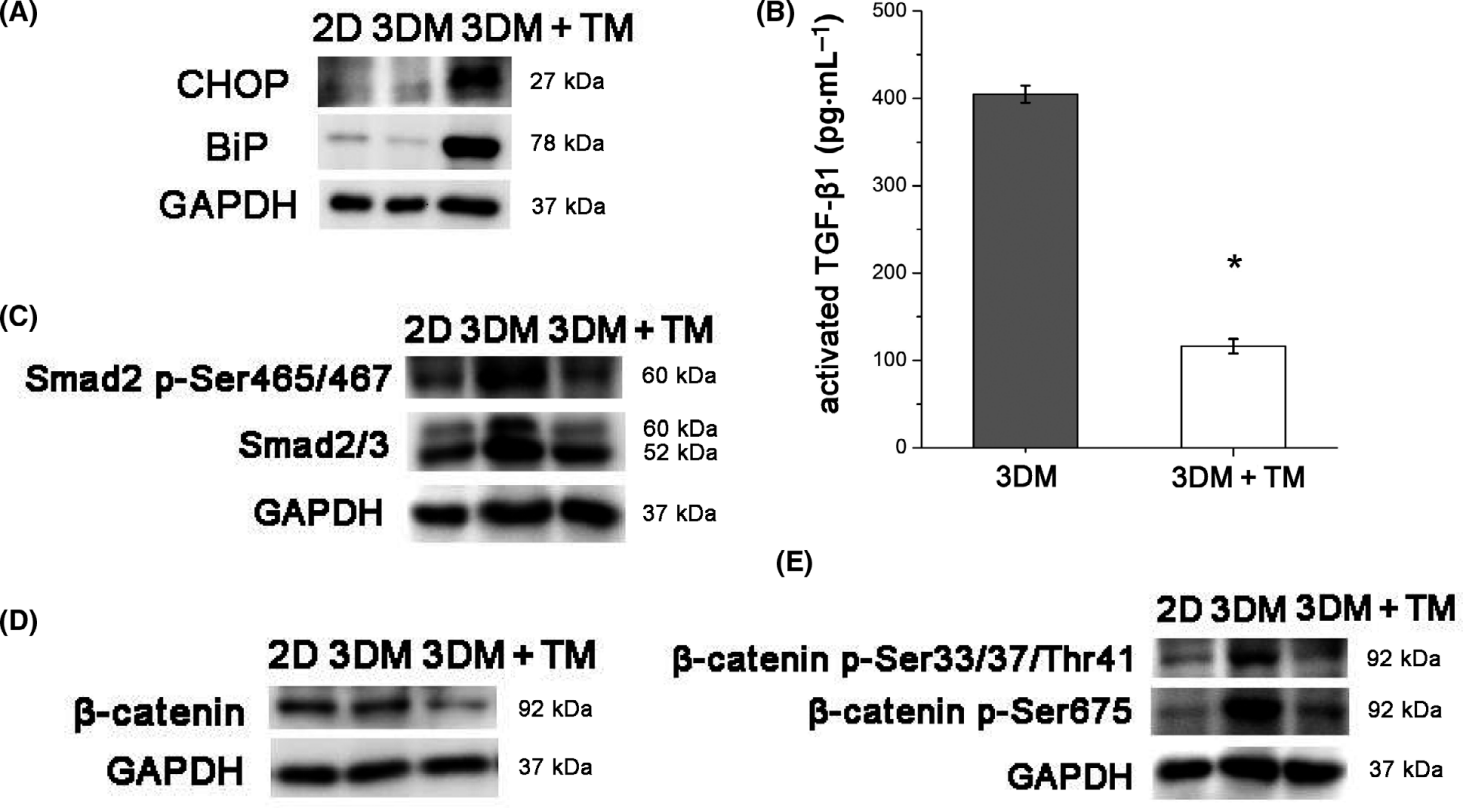
TM‐induced ER stress activation and the changes of TGF‐β1/Smad2/3 and β‐catenin signaling. (A) Western blot analysis of Bip and CHOP of MDA‐MB‐231 cells in 2D, 3DM and 3DM+TM. (B) Concentrations of activated TGF‐β1 of MDA‐MB‐231 cells in 3DM and 3DM+TM (*P < 0.05). Statistical significance was determined for experimental data by using the Student's *t*‐test. Error bars indicate SEM. All experiments were repeated three times. (C) Western blot analysis of Smad2/3 and Phospho‐Smad2 of MDA‐MB‐231 cells in 2D, 3DM and 3DM+TM. (D, E) Western blot analysis of total β‐catenin, active β‐catenin (p‐Ser675) and inactive β‐catenin (p‐Ser33/37/Thr41) in 2D, 3DM and 3DM+TM.

To investigate the effect of TM by activating the ER stress signaling pathway, we performed western blot analysis to detect the expression levels of Bip and CHOP in 3DMs after TM treatment. As shown in Fig. 4A, the protein expression levels of Bip and CHOP were significantly elevated in cells treated with TM, indicative of ER stress activation. The expression of other ER stress‐induced genes XBP‐1 and IRE1 after inducing ER stress in MDA‐MB‐231 cells was also up‐regulated (Fig. S2A,B).

### Involvement of TGF‐β1/Smad2/3 and β‐catenin signaling in VM

We studied the concentrations of activated TGF‐β1 in 3D cultured cells before and after TM treatment and found that activated TGF‐β1 significantly declined in 3D cultures after TM treatment (*P < 0.05) (Fig. 4B). Western blots revealed that downstream Smad2/3 and Phospho‐Smad2 increased markedly in 3DMs compared with 2D cultures and decreased significantly after TM treatment, indicating the inhibition of the TGF‐β1 signaling that was activated in 3D Matrigel before TM treatment (Fig. 4C).

We studied the changes of β‐catenin in 3DM cells before and after TM treatment. As shown in Fig. 4D, western blot analysis revealed that compared with MDA‐MB‐231 cells in 3D Matrigel with VM phenotype, cells after TM treatment that lost their VM phenotype exhibited significant down‐regulation of total β‐catenin. Then we studied the expression of active β‐catenin (p‐Ser675) and inactive β‐catenin (p‐Ser33/37/Thr41), both of them significantly down‐regulated after TM treatment (Fig. 4E).

### β‐Catenin knockdown suppresses VM

**Fig. 5:**
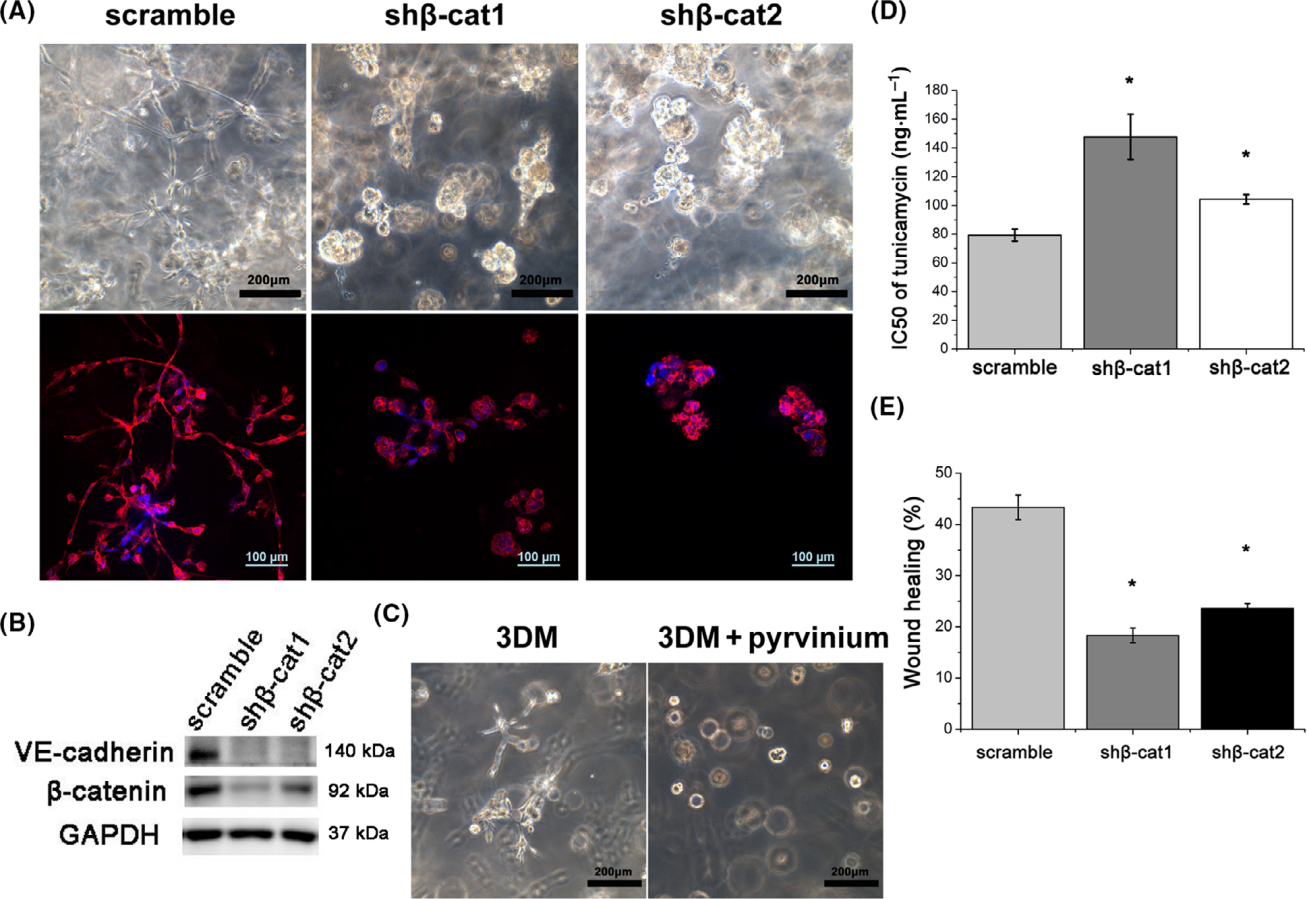
The effects of β‐catenin knockdown on the formation of VM and cellular behaviors. (A) β‐Catenin knockdown inhibited the VM phenotype and reduced the tubular networks of MDA‐MB‐231 cells in 3DM. Scale bars: 200 μm (top); 100 μm (bottom). (B) Western blot analysis of VE‐cadherin of scramble control and β‐catenin knockdown (shβ‐cat1 and shβ‐cat2) groups. (C) Pyrvinium significantly reduced VM formation of MDA‐MB‐231 cells in 3DMs. Scale bars: 200 μm. (D) IC_50_ of 2D cultured MDA‐MB‐231 cells to TM after β‐catenin knockdown (*P < 0.05). (E) Wound healing assays revealed that knockdown of β‐catenin inhibited cells migration (*P < 0.05). Statistical significance was determined for experimental data by using the Student's *t*‐test. Error bars indicate SEM. All experiments were repeated three times.

To further explore the effects of β‐catenin signaling on the formation of VM, we conducted shRNA knockdown of β‐catenin in MDA‐MB‐231 cells. We observed that knockdown of β‐catenin strikingly reduced the VM formation of MDA‐MB‐231 cells in 3DMs and reduced the number of branch points and patterned tubular networks (Fig. 5A), accompanied by down‐regulation of VE‐cadherin (Fig. 5B). Moreover, treatment of 3DM MDA‐MB‐231 cells with 50 nm pyrvinium, a potent inhibitor of the β‐catenin signaling pathway [33], also significantly reduced VM formation in 3DMs (Fig. 5C). Different concentrations of TM treatment revealed that IC_50_ of 2D cultured MDA‐MB‐231 cells after β‐catenin knockdown significantly elevated compared with the scramble control (*P < 0.05) (Fig. 5D). The results indicated that β‐catenin knockdown enhanced the resistance of MDA‐MB‐231 cells to TM and might suppress TM‐induced apoptosis via resolution of ER stress. To investigate the role of β‐catenin knockdown on MDA‐MB‐231 cells migration, we applied wound healing assays (Fig. S2C) to study the migration ability, and the data indicated β‐catenin knockdown significantly decreased the migratory behaviors of MDA‐MB‐231 cells (Fig. 5E).

## Discussion

In this study, we investigated the effects of ER stress on the VM phenotype of aggressive TNBC MDA‐MB‐231 cells and explored the involvement of TGF‐β1/Smad2/3 and β‐catenin signaling in it.

VM was observed in metastasis associated with aberrant extravascular expression of VE‐cadherin [7]. It is still not clear why tumor cells acquire the phenotype and express nonendothelial VE‐cadherin. In our study, we investigated 3DMs of several breast cancer cells of MDA‐MB‐231, BT549, MDA‐MB‐435S and MCF‐7, and one nonmalignant breast cell of MCF 10A. The results revealed that MDA‐MB‐231 and BT549 cells with mesenchymal phenotype could form tubular networks in 3DMs, while only MDA‐MB‐231 cells expressed VE‐cadherin. Although it has been reported that TNBC BT‐549 cells could efficiently undergo matrix‐associated VM formation in the 3D Matrigel cell culture model [34]. Because VE‐cadherin is one of the most important markers for VM phenotype, we considered that BT‐549 cells formed tubular networks in 3DMs instead of VM.

TM has been widely used to study the effects of glycosylation on metastasis progression and sensitivity of drugs with antitumor activity to tumor cells [35, 36, 37]. It has been reported that TM‐induced ER stress could reduce *in vitro* subpopulation and invasion of CD44^+^/CD24^−^ phenotype breast cancer stem cells [38], which indicated the relationship among deglycosylation, ER stress and stemness of cancer cells. VM is one kind of phenotype of aggressive tumor cells, especially those with properties of stemness and EMT [2, 3, 4, 5], which mimic vasculogenesis by forming patterned tubular networks in the tumor ECM. Because the key ER stress signaling pathway affects tumor angiogenesis and progression via the microenvironment [23, 24, 25], we proposed a hypothesis that there are relationships between ER stress and VM in TNBC, a highly aggressive malignancy with poor posttreatment prognosis, and decided to explore the effects of TM‐induced ER stress on VM phenotype and related markers and signaling pathways involved in it.

After TM treatment, Bip and CHOP were significantly elevated in cells treated with TM indicative of the ER stress activation. MDA‐MB‐231 cells in 3D Matrigel formed tumor spheroid morphology instead of VM phenotype. Because both TM and thapsigargin are used as common ER stress inducers, we checked the effects of thapsigargin on VM. The result showed that thapsigargin was able to inhibit VM phenotype similar to TM, implying that the inhibition of VM phenotype by induction of ER stress was not a TM‐specific effect but might be a general result. Meanwhile, the expression of VE‐cadherin and Bmi‐1 was significantly down‐regulated. Loss of VE‐cadherin demonstrated the VM phenotype formation was destroyed by TM‐induced ER stress. The decrease of Bmi‐1 expression and proportion of CD44^+^/CD24^−^ subpopulation cells showed the loss of stemness and that TM‐induced ER stress might result in differentiation of cancer stem cells. Moreover, activated TGF‐β1 significantly declined in 3D cultures after TM treatment. Smad2/3 are critical downstream regulators of TGF‐β1 signaling pathway, and the phosphorylated Smad2/3 regulates gene transcription in nucleus. Smad2/3 and Phospho‐Smad2 decreased significantly after TM treatment, indicating the inhibition of the TGF‐β1 signaling. Furthermore, total β‐catenin, active β‐catenin (p‐Ser675) and inactive β‐catenin (p‐Ser33/37/Thr41) significantly down‐regulated after TM treatment. β‐Catenin signaling was reported to be involved in the formation of VM phenotype [13, 14], and the extracellular domain of VE‐cadherin could form a zipper‐like structure between cells, while the intracellular domain interacts with β‐catenin [39]. The immunostaining study of VE‐cadherin and β‐catenin in 3D cultured MDA‐MB‐231 cells with VM phenotype showed that VE‐cadherin and β‐catenin colocalized in the cell surface of the VM tubular structure (Fig. S2D). Then we conducted shRNA knockdown of β‐catenin, which resulted in the significantly reduced VM formation in 3D matrigel cultures. It has been reported that the UPR is required for the definitive endodermal specification of mouse embryonic stem cells via Smad2 and β‐catenin signaling [40]. Our study further proved that ER stress could inhibit 3D Matrigel‐induced VM of breast cancer cells via TGF‐β1/Smad2/3 and β‐catenin signaling. Moreover, we examined the effects of VEGFA on VM phenotype. The results indicated that after TM treatment, the expression of VEGFA was down‐regulated. Knockdown of VEGFA in MDA‐MB‐231 cells resulted in significant phenotype changes (the cellular networks of VM vs. decreased branching and tumor spheroid morphology) similar to that in TM‐treated cells (Fig. S2E).

Cancer‐associated glycoproteins exhibit aberrant glycosylation, including mucins, integrins and cadherins [41]. However, the relationship between glycosylation and VM is not clear. As the largest subgroup of integrins, integrin β1 is the central node of ECM signal transduction [42, 43] and an important mediator of breast cancer progression [44]. Moreover, integrin β1 has been shown to play a critical role in the formation of cell network structures of VM in 3D high‐density collagen [45]. Altered glycosylation of integrin β1 is prevalent in tumor cells accompanying phenotypic changes and is associated with cell invasiveness and metastasis [46]. In 3DMs, MDA‐MB‐231 formed the VM phenotype, which indicated full glycosylation of integrin β1. After treatment of TM, a N‐glycosylation inhibitor, the glycosylation status of the integrin β1 was changed, along with the ER stress activation, changes of cellular phenotype and activated TGF‐β1, exhibiting a series of bands of incompletely glycosylated peptide. The conversion of the 115‐kD integrin β1 precursor into the mature 130‐kD form was mediated by TGF‐β1 [30], hence their crosstalk needs to be further explored in the future.

In conclusion, our study revealed that the activation of ER stress inhibited the VM phenotype of breast cancer cells via both the TGF‐β1/Smad2/3 and β‐catenin signaling pathways. Future exploration of mechanisms involved in ER stress and VM in breast cancer could lead to more precise target therapies to inhibit the vessels formation that might complement the insufficient efficacy of antiangiogenic therapy in TNBC.

## Conflict of interest

The authors declare no conflict of interest.

## Author contributions

HW, JH and KM conceived and designed the experiments. HL, HW, DC and CG performed the experiments and acquired and analyzed data. HL and HW wrote the manuscript. KM reviewed and edited the manuscript.

## Supporting information

**Fig. S1.** (A) Phenotype of MCF 10A, MCF‐7, MDA‐MB‐435S, MDA‐MB‐231 and BT‐549 cells in 3D Matrigel cultures. Scale bars: 50μm. (B) Expression of VE‐cadherin in tubular networks of BT‐549 and MDA‐MB‐231 cells. (C) Phenotype of MDA‐MB‐231 in 2D and 3D collagen culture (3DC). Scale bars: 200μm.

**Fig. S2.** (A) Western blot analysis of XBP‐1 of MDA‐MB‐231 cells in 2D, 3DM and 3DM+TM. (B) Western blot analysis of IRE1 of MDA‐MB‐231 cells in 2D, 3DM and 3DM+TM. (C) Representative photographic images of wound healing assays of scramble control, and β‐catenin knockdown (shβ‐cat1 and shβ‐cat2) groups. Scale bar: 100μm. (D) Immunostaining images of VE‐cadherin (green), β‐catenin (red) and nuclear (blue) DAPI in 3D cultured MDA‐MB‐231 cells with VM phenotype. Scale bars: 100μm. (E) The effects of TM on VEGFA expression and VEGFA knockdown on the VM phenotype. Scale bars: 200μm.

## Data Availability

The data are available from the corresponding author upon reasonable request.
