# Supplementary figures and images for: Endoplasmic reticulum stress inhibits 3D Matrigel‐induced vasculogenic mimicry of breast cancer cells via TGF‐β1/Smad2/3 and β‐catenin signaling
Source: FEBS Open Bio. 2021 Aug 19;11(9):2607–18. doi: 10.1002/2211-5463.13259 (PMC8409287; doi:10.1002/2211-5463.13259)

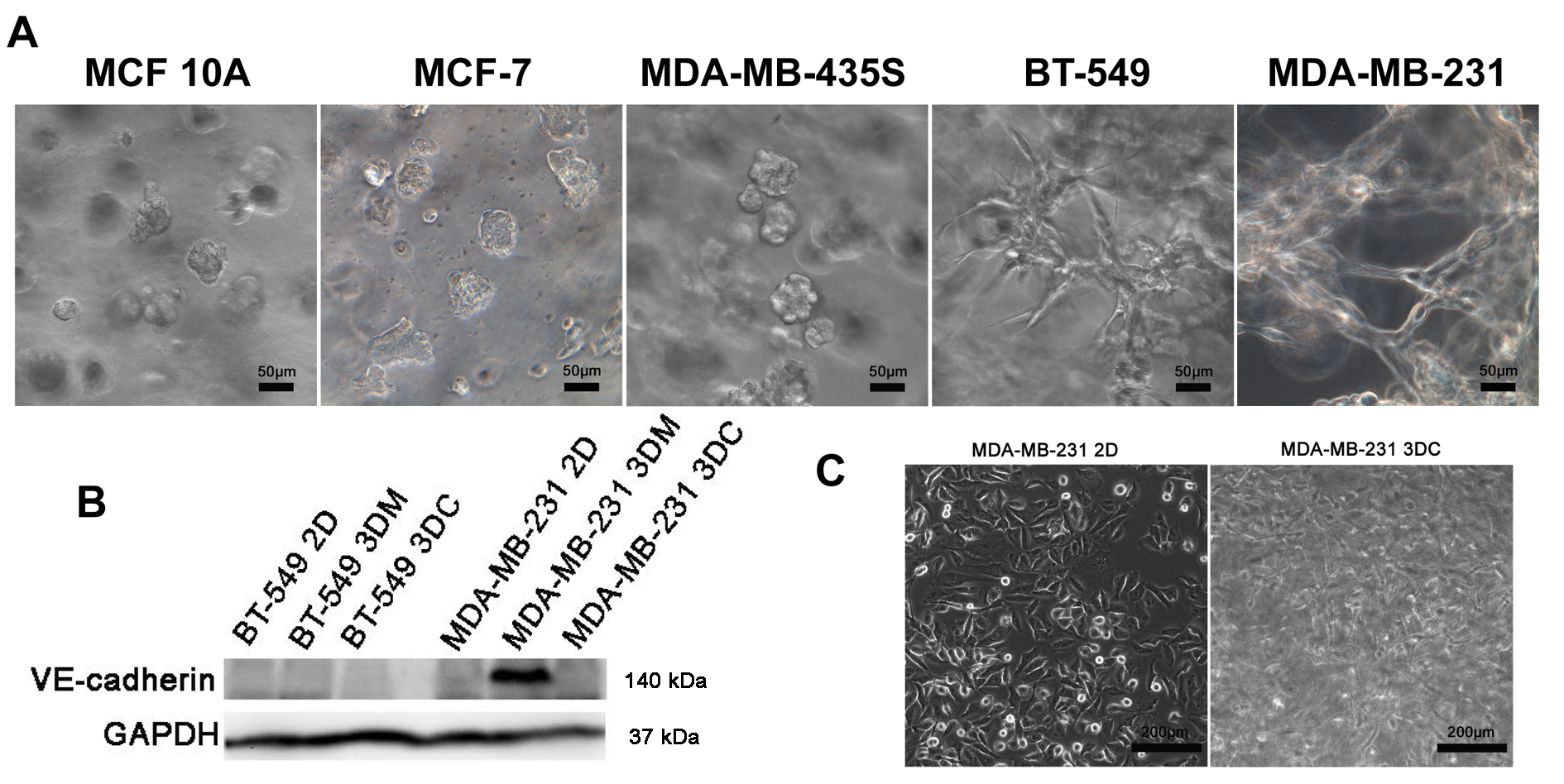

Supplement: Supplementary file 1 — Fig. S1. (A) Phenotype of MCF 10A, MCF‐7, MDA‐MB‐435S, MDA‐MB‐231 and BT‐549 cells in 3D Matrigel cultures. Scale bars: 50μm. (B) Expression of VE‐cadherin in tubular networks of BT‐549 and MDA‐MB‐231 cells. (C) Phenotype of MDA‐MB‐231 in 2D and 3D collagen culture (3DC). Scale bars: 200μm. [file FEB4-11-2607-s002.tif]

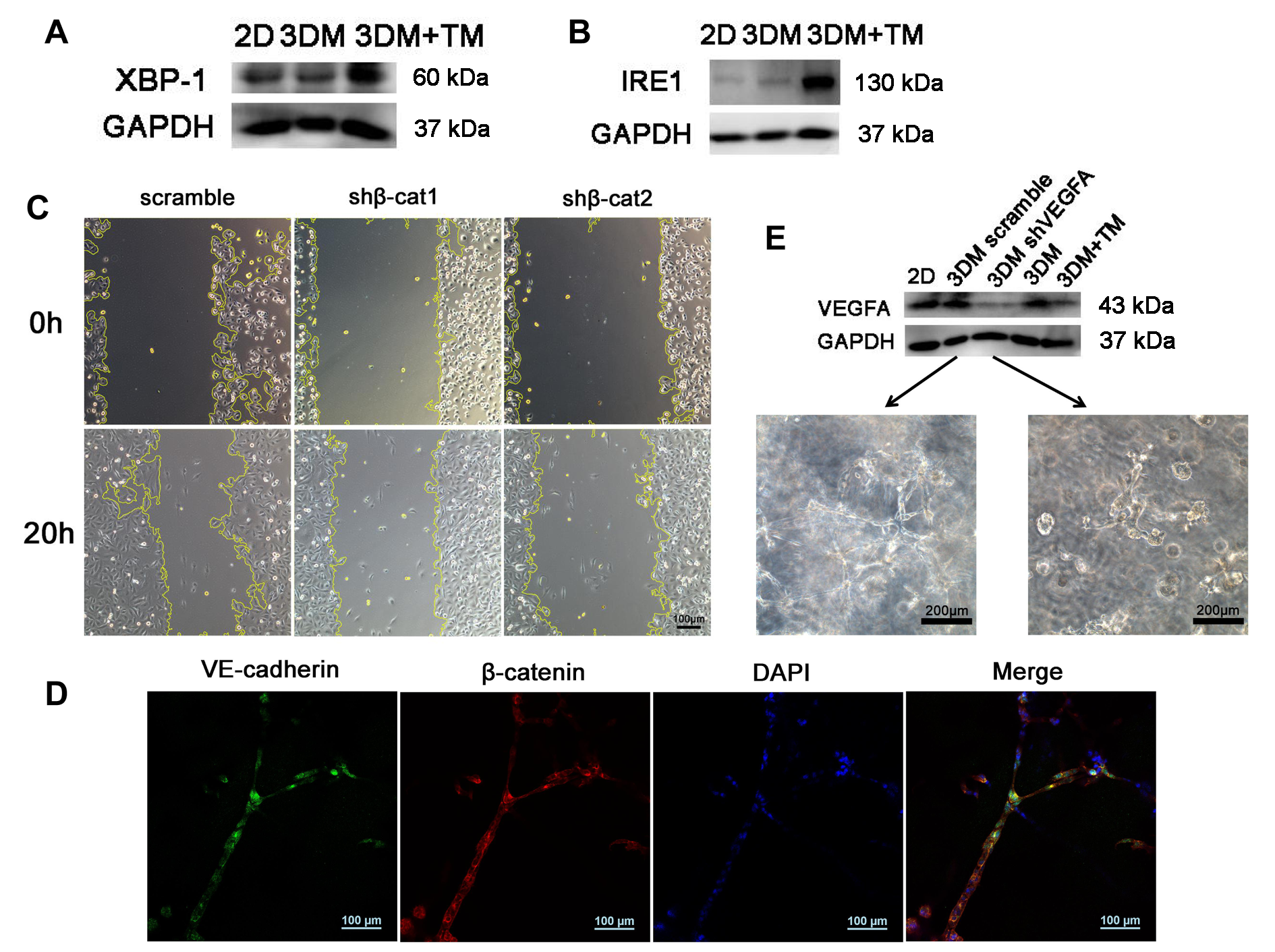

Supplement: Supplementary file 2 — Fig. S2. (A) Western blot analysis of XBP‐1 of MDA‐MB‐231 cells in 2D, 3DM and 3DM+TM. (B) Western blot analysis of IRE1 of MDA‐MB‐231 cells in 2D, 3DM and 3DM+TM. (C) Representative photographic images of wound healing assays of scramble control, and β‐catenin knockdown (shβ‐cat1 and shβ‐cat2) groups. Scale bar: 100μm. (D) Immunostaining images of VE‐cadherin (green), β‐catenin (red) and nuclear (blue) DAPI in 3D cultured MDA‐MB‐231 cells with VM phenotype. Scale bars: 100μm. (E) The effects of TM on VEGFA expression and VEGFA knockdown on the VM phenotype. Scale bars: 200μm. [file FEB4-11-2607-s001.tif]
